# Factors affecting the periapical healing process of endodontically treated teeth

**DOI:** 10.1590/1678-7757-2016-0464

**Published:** 2017

**Authors:** Roberto Holland, João Eduardo Gomes, Luciano Tavares Angelo Cintra, Índia Olinta de Azevedo Queiroz, Carlos Estrela

**Affiliations:** 1 Universidade Estadual Paulista, Faculdade de Odontologia de Araçatuba, Departamento de Odontologia Restauradora, Araçatuba, SP, Brasil; 2 Universidade Federal de Goiás, Faculdade de Odontologia, Departamento de Ciências Estomatológicas, Goiânia, GO, Brasil

**Keywords:** Root canal therapy, Periapical periodontitis, Endodontics, Clinical protocols

## Abstract

Tissue repair is an essential process that reestablishes tissue integrity and regular function. Nevertheless, different therapeutic factors and clinical conditions may interfere in this process of periapical healing. This review aims to discuss the important therapeutic factors associated with the clinical protocol used during root canal treatment and to highlight the systemic conditions associated with the periapical healing process of endodontically treated teeth. The antibacterial strategies indicated in the conventional treatment of an inflamed and infected pulp and the modulation of the host's immune response may assist in tissue repair, if wound healing has been hindered by infection. Systemic conditions, such as diabetes mellitus and hypertension, can also inhibit wound healing. The success of root canal treatment is affected by the correct choice of clinical protocol. These factors are dependent on the sanitization process (instrumentation, irrigant solution, irrigating strategies, and intracanal dressing), the apical limit of the root canal preparation and obturation, and the quality of the sealer. The challenges affecting the healing process of endodontically treated teeth include control of the inflammation of pulp or infectious processes and simultaneous neutralization of unpredictable provocations to the periapical tissue. Along with these factors, one must understand the local and general clinical conditions (systemic health of the patient) that affect the outcome of root canal treatment prediction.

## Introduction

The process of healing depends on the structural and functional replacement of the areas affected by intrinsic or extrinsic factors. It can involve the repair and regeneration of the affected site^18^. Additionally, inflammation is a defense mechanism that occurs in vascularized tissue. Connective tissue involves a process of repair via the formation of granulation tissue, whereas non-connective tissue, such as that of the glandular organs, smooth muscles, skeletal muscles and peripheral nerves, involves the proliferation, and therefore, regeneration, of the remaining tissue^18^. These two processes are dependent upon the regenerative capacity of the affected cells, the extent of the affected site, and the proliferative activity of the stromal tissue. Regeneration involves a process of tissue renewal with cells that have similar characteristics to those that were previously lost; it is the morphological and functional restoration of tissue. Conversely, repair is characterized by the formation of connective tissue at the site of the lesion, and the infiltration of fibroblast. The process of healing begins with inflammation, and is resolved by the clearance of the immunogen that induces the tissue response^10^.

Complete repair only occurs once the antigen has been neutralized during the inflammatory response. During pulp infection, the occluded blood supply of the root canal becomes conducive to bacterial proliferation. Furthermore, inflammation within the periapical region is elicited to neutralize the antigen. Bone resorption is also stimulated by this inflammatory response, and space is created for the infiltration of immune cells, which are then organized into a barrier to sequester the infection.

Bone resorption and bone formation are processes involving the activity of osteoclasts, osteoblasts, and osteocytes; they are affected by the systemic and local conditions^66^. However, bone homeostasis is disrupted during apical periodontitis, which promotes increased rates of bone resorption.

Ideally, cases of apical periodontitis should be characterized by a repair process that is asymptomatic in nature, an absence of radiographic abnormalities in the periradicular tissues, and evidence of biological sealing (cement sealing the foramina), or by the presence of a fibrous capsule with the infiltration of few inflammatory cells^54^. Thus, the application of clinical protocols should consider the local and systemic conditions that contribute towards the healing process.

This review aims to discuss the important therapeutic factors underlying the clinical protocols used during a root canal treatment. Additionally, we will analyze local and systemic conditions associated with the periapical healing process of endodontically treated teeth.

### Therapeutic factors that affect the repair process

#### Biochemical preparation

Root canal shaping is an important step in endodontic therapy to achieve the apical healing and the cleaning and modeling of the root canal system. However, the complex root canal anatomy, associated with the presence of curvatures and ramifications, the shape, and the position of the apical foramina, can interfere and hinder the root canal shaping and cleaning^79^.

The advances in endodontic therapy, specifically in the development of new instruments made of nickeltitanium (NiTi) alloys (rotatory and reciprocating files) - with characteristics such as superelasticity (flexibility) and shape memory –, simplified the cleaning and shaping of root canals by reducing the instrumentation time and by keeping the original canal shape^5^ ^,^ ^35^ ^,^ ^88^. Besides, reduction of the occurrence of zips, ledges, perforations, and transporting of the root canals, especially in narrow and curved canals^35^ ^,^ ^88^, were also observed after using those instruments. Despite all these improvements, the possibility of fracture of NiTi instruments, mainly in root canals with severe curvatures, has been found^76^ ^,^ ^82^. Moreover, Panitvisai, et al.^75^ (2010) showed no reduction on the prognostic of endodontic treatment when a fractured instrument fragment is left within a root.

Investigations comparing the manual, rotary, and reciprocating systems and the association between them have been extensively performed^5^ ^,^ ^35^ ^,^ ^88^. Azar, et al.^4^ (2012) reported no significant differences in cleaning efficiency between manual and rotary instruments. Besides, evaluation of the apical bacterial extrusion promoted by reciprocating and rotary instrumentation showed that both systems extruded bacteria beyond the foramen; nevertheless, the reciprocating showed lesser extrusion than the rotary system^107^. Additionally, no differences in the efficacy of rotary and reciprocating systems for removing root filling material was identified^93^. However, both systems in association were able to remove a large amount of root filling material in the retreatment of curved canals^84^.

Subramaniam, et al.^97^ (2013) showed that both manual and rotatory instrumentation were able to reduce the aerobic and anaerobic microflora of root canals of primary molars. Meanwhile, bacteria were detected in root canals of teeth with apical periodontitis before and after the use of hand and rotary NiTi instrumentation and no difference in bacterial reduction in infected canals after using both instruments was observed^83^. Furthermore, Pinheiro, et al.^80^ (2012) compared the cleaning effectiveness of manual, hybrid, and rotary instrumentation to remove *Enterococcus faecalis* from primary molar teeth. The authors found that all techniques were able to reduce the number of *Enterococcus faecalis;* thus, hybrid instrumentation showed to better reduction when compared with manual.

Therewith, even with the improvement on the biomechanical preparation of root canal system using NiTi instruments, bacteria can survive and grow inside root canal systems or on apical biofilm^47^ ^,^ ^83^ ^,^ ^105^, compromising the periapical tissue repair. Therefore, the complete repair depends on the association of an effective irrigating solution, intracanal dressing, and root canal filling to the mechanical preparation.

#### Irrigating solutions

Root canal treatment (RCT) requires the use of irrigating solutions to provide an antimicrobial effect, remove debris, and neutralize the presence of organic compounds. Because of the risk of periapical extrusion via the apical foramen, irrigating solutions should be biocompatible and non-irritating to the periapical tissue^37^ ^,^ ^58^ ^,^ ^94^_._

The persistence of bacterial infection following root canal preparation reveals the limitations of the irrigating solutions, such as sodium hypochlorite (NaOCl) and chlorhexidine (CHX). These solutions can only reduce the microbial population and, therefore, cannot entirely eliminate it. The sanitization process consists of the disinfection and enlargement of the root canal via the action of sodium hypochlorite and the instrumentation techniques of the root canal, respectively. Furthermore, these protocols reduce the remaining microbiota, which improves the efficacy of the intracanal dressing and increases the success rate of the endodontic treatment^22^.

The efficacy of NaOCl and CHX on the incidence of *Enterococcus faecalis* infection was discussed in a systematic review and meta-analysis^23^. Sodium hypochlorite is a commonly used endodontic irrigant because of its antibacterial properties and its ability to dissolve organic tissue^21^ ^,^ ^50^ ^,^ ^103^.

The antibacterial action of NaOCl can be verified based on its physicochemical properties and its reaction with organic tissue^21^ ^,^ ^50^. Sodium hypochlorite acts as a solvent for organic compounds and lipids; it degrades fatty acids into the products of fatty acid salts (soap) and glycerol (alcohol), in a saponification reaction. This reduces the surface tension of the remaining solution. Furthermore, NaOCl is able to cause amino acids to undergo a neutralization reaction to form water and salt. Similarly, hypochlorous acid (HOCl) acts as solvent in the presence of organic tissue, and releases chlorine; this combines with the amine group of the protein and forms chloramines, in a chlorination reaction. Hypochlorous acid and hypochlorite can undergo a reaction with amino acids, which would result in their degradation and hydrolysis. The chlorination reaction between chlorine and the amine group (-NH_2_) results in the product of chloramines, which interfere with cell metabolism. Chlorine is a strong oxidant; it has antimicrobial properties, and is able to inhibit bacterial enzymes. This results in the irreversible oxidation of sulfhydryl (-SH) group, which is essential for the function of bacterial enzymes^21^ ^,^ ^50^.

Frough-Reyhani, et al.^26^ (2016) evaluated the antimicrobial activity of 1%, 2.5%, and 5% NaOCl solution in the treatment of *Enterococcus faecalis* biofilms at different stages of development and showed that 2.5% and 5% NaOCl completely eliminated the biofilms in three stages of development, whereas the bacteria in mature and old biofilms were more resistant to 1% NaOCl. In an *in vitro* study, Siqueira, et al.^95^ (2000) found no difference in the efficacy of the antibacterial activity of 1%, 2.5%, and 5% NaOCl solution. Moreover, regardless of the concentration, NaOCl was also ineffective in the removal of the smear layer formed during root canal preparation; the smear layer impairs the cleaning of the root canal system^59^. Reinfection of the root canal system after instrumentation has been reported^83^. Teeth with periapical lesions and a higher concentration of endotoxins have also been detected^36^. Sodium hypochlorite solutions can inhibit the action of certain endotoxins; however, it is not effective against all of them^74^.

The smear layer is the material that is attached to the canal walls during root canal preparation. It is composed of dentin, remnants of pulp tissue (organic and inorganic components), chemical residues, and microorganisms. The use of EDTA and NaOCl solutions has been shown to be more effective to remove the smear layer^104^.

Nery, et al.^73^ (2011) reported similar results following the use of 1% and 2.5% NaOCl solution during the endodontic irrigation of canine teeth. The incidence of pulp necrosis and apical periodontitis was reported and treated in one session. Therefore, the use of 1% and 2.5% NaOCl solution had similar treatment efficacy. Nonetheless, the use of 2.5% NaOCl solution is preferred for clinical purposes because of its more stable nature.

Recently, new antimicrobial approaches to disinfection of root canals have been proposed, such as photodynamic therapy (PDT), a therapy that combines the association between a photosensitizer dye and a specific wavelength light Laser (light amplification by stimulated emission of radiation) or Led (light emitting diode). The singlet oxygen, which is responsible for the disruption of the bacterial membrane and inactivation of endotoxins, promotes antimicrobial action^67^ ^,^ ^92^. Several investigations have been reporting that PDT reduces the number of bacteria from root canal^30^ ^,^ ^61^ ^,^ ^108^ and shows the same efficacy of 2.5% and 5% NaOCl solution to eliminate *Enterococcus faecalis* ^114^ ^,^ ^115^. In addition, Vaziri, et al.^110^ (2012) found that the combination of PDT and 2.5% NaOCl solution was more effective against *Enterococcus faecalis.* Moreover, Borsatto, et al.^7^ (2016) evaluated the response of the apical and periapical tissues of canine teeth with apical periodontitis after one session with and without antimicrobial photodynamic therapy (aPDT) and compared it with two sessions using calcium hydroxide as intracanal dressing. They showed that the better repair and small periapical lesions were associated with the two sessions using calcium hydroxide.

Thus, despite all of these new antimicrobial methods, NaOCl solutions are still the greatest choice for clinical procedure due to their effective antimicrobial potential. Moreover, because of the instability of these solutions, especially during storage and transportation time, the 1% NaOCl solution may not be effective; thus, the 2.5% NaOCl solution is the most recommended.

### Intracanal dressing

Several types of medication have been developed and used as intracanal dressing^51^. However, the efficacy of some treatments has been questioned; this has contributed towards the increasing preference among professionals to perform RCT in a single session^34^ ^,^ ^68^ ^,^ ^77^ ^,^ ^81^. Additionally, the practice of single-appointment endodontics, as opposed to multiple-appointment endodontics, has been encouraged by the negligible differences in the treatment outcomes between the two, which include an absence of postoperative pain and the clinical and radiographic repair^77^ ^,^ ^78^ of teeth. Conversely, studies assessing canine teeth have reported superior results with the practice of multiple-appointment endodontic treatment^54^ ^,^ ^64^.

In an attempt to further clarify this issue, Holland, et al.^54^ (2003) conducted a study using canine teeth as a model. These specimens were characterized by a necrotic pulp and had periapical lesions that were treated in either one or two appointments. The teeth that were treated in two appointments received a course of calcium hydroxide intracanal dressing for 7 or 14 days. The root canals were filled with Sealapex and were evaluated for six months after the treatment. The authors observed that the use of calcium hydroxide for 7 or 14 days obtained better results than the single-appointment endodontic treatment. Furthermore, they attributed the repair process observed after the single appointment to the alkaline pH of Sealapex; once the root canals were filled with Sealapex, it showed better results than the other sealers that were used in teeth with apical periodontitis^63^ ^,^ ^64^.

Georgopoulou, et al.^33^ (1993) reported that the calcium hydroxide intracanal dressing was more effective against anaerobic bacteria than the use of camphorated paramonochlorophenol. The high pH of calcium hydroxide, due to the release of hydroxide ions, is capable of altering the structural integrity of the cytoplasmic membrane of bacteria^24^. Additionally, the calcium hydroxide had an indirect effect on the anaerobic microorganisms of the root canal because of the reaction between calcium ion and aqueous carbon dioxide^24^. Calcium hydroxide also results in the degradation of bacterial lipopolysaccharides^1^ ^,^ ^24^.

The biological and antimicrobial action of calcium hydroxide is based on its dissociation into calcium and hydroxide ions, and on the action of these ions on tissues and bacteria^24^. Calcium hydroxide induces the deposition of a hard tissue bridge on the pulpal and periodontal connective tissue^54^. Its action on connective tissue (pulpal and periodontal tissues) stimulates mineralization from the significant involvement of alkaline phosphatase and fibronectin^54^ ^,^ ^71^.

Nair, et al.^72^ (2005) evaluated the intracanal microbial status of the mandibular first molars with primary apical periodontitis, in humans, following single-appointment endodontic treatment. The results showed the anatomical complexity of the root canal system of the mandibular first molar, and highlighted the ineffectiveness of contemporary instruments and irrigation processes in the removal of local flora after only one appointment. This was mostly attributable to their organization as biofilms in inaccessible areas of the canal system. Therefore, to achieve a highly favorable long-term prognosis of the RCT, the stringent application of all nonantibiotic and chemomechanical measures must be taken to treat teeth with infected and necrotic root canals; additionally, these measures are required to disrupt the formation of biofilms and to minimize the intraradicular microbial load.

The challenges affecting a successful root canal preparation include the following factors: complex anatomy; number of canals; curvatures; root canal ramifications; shape; and position of apical foramina, which complicates cleaning^22^ ^,^ ^79^. Therewith, to achieve a complete root canal cleaning, an intracanal dressing must be used. Thus, the biological properties of calcium hydroxide, as well as its antimicrobial capacity to induce the deposition of a hard tissue, promoting a better repair, make it the intracanal medication recommended during the RCT.

### Root canal filling

The goal of root canal obturation is to obtain complete sealing in order to hinder the communication between the root canal and the periapical tissue. This favors the process of apical and periapical repair after endodontic therapy^53^.

An ideal root canal sealer must have adequate physical, chemical, and biological properties^31^ ^,^ ^86^. Several factors can affect the success of RCT, including the type and composition of sealer utilized^31^ ^,^ ^57^ ^,^ ^86^. The presence and release of substances from the sealers can cause different reactions when in contact with tissue^86^.

**Figure 1:**
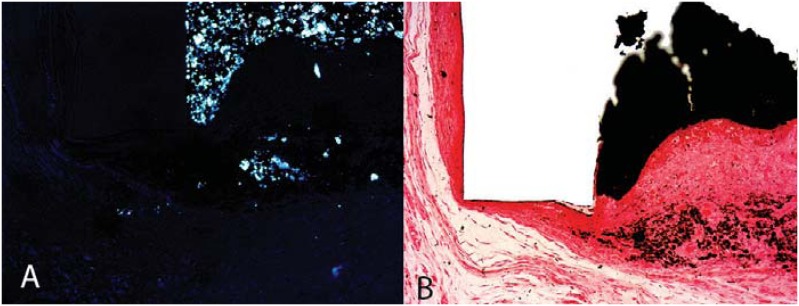
(A) Presence of dystrophic calcification on the tube opening; (B) Presence of birefringent structures exposed by polarized light; they have been formed by the reaction between the calcium ions derived from the material and the carbonic gas released from the tissues (Sealapex, 30 days, Von Kossa and polarized light; 10×)

The reaction between calcium oxide and tissue fluid was previously described^55^. This reaction produces calcium hydroxide, which dissociates into calcium and hydroxide ions in the presence of water. Calcium ions then react with aqueous carbon dioxide within the tissue to form crystals of calcium carbonate, which subsequently stimulate the deposition of hard tissue (Figure 1). Therefore, root canal sealers that contain calcium hydroxide or calcium oxide have been clinically used.

The tissue response to Sealapex, CPM Sealer, and MTA Fillapex, which contain in their composition calcium hydroxide or calcium oxide, were evaluated in the subcutaneous tissue of rats. The results showed that all sealers induced a moderate inflammatory response at 7 and 15 days, which resolved over time. In addition, all sealers were capable of stimulating the deposition of mineralized tissue^42^ ^,^ ^44^. Another study^43^ evaluated the efficacy of RCT using the aforementioned sealers, in canine teeth, following a single session of endodontic treatment. Incomplete periapical tissue repair was reported, and the sealers were capable of controlling the root canal infection regardless of the differences in their antimicrobial activity^20^ ^,^ ^101^. The use of Sealapex and MTA Fillapex resulted in a similar seal and had a better response than the use of CPM Sealer. This was probably due to the fact that both sealers produced less of an inflammatory reaction, therefore showing their biocompatible nature^40^.

Furthermore, even with the use of biocompatible sealers, which are capable of inducing mineralization, complete repair is only possible with the disinfection of the root canal system. Therefore, an improved repair process following RCT is promoted by the complete cleaning of the root canal, as well as by the use of an intracanal dressing, such as calcium hydroxide.

### Apical limit of obturation

Systematic reviews have shown that root canal preparation and obturation inferior to the radiographic apex (root canal obturation at 1-2 mm inferior to the apex) were associated with a better prognosis (higher success rates)^87^.

**Figure 2:**
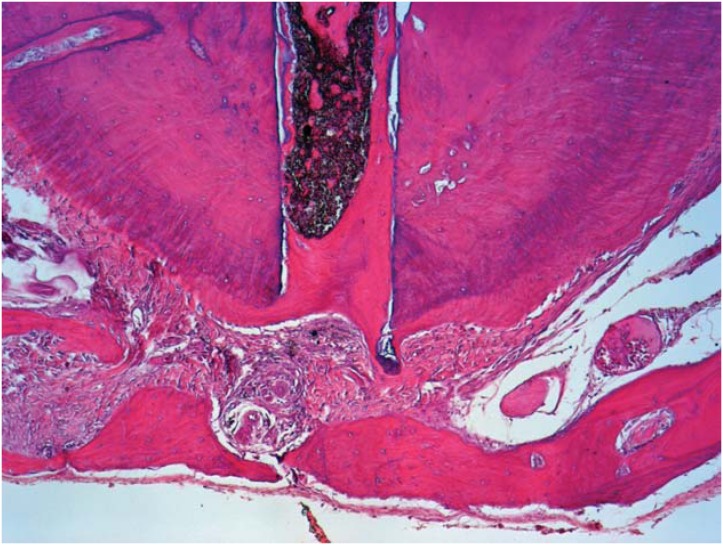
Process of obturation from the apical limit; a new cement sealing technique in the apical foramen, in close contact with Sealapex. Observe the normal organization of the periodontal ligament and alveolar bone close to the periapex (HE, 10×)

**Figure 3:**
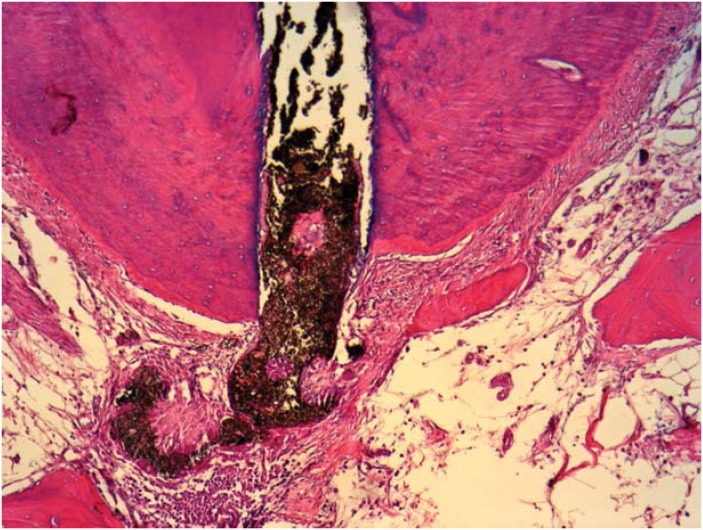
Obturation over the apical limit showing the absence of new cement sealing the apical foramen in close contact with Sealapex. Observe the partial disorganization of the periodontal ligament with the chronic inflammatory cells surrounding the extruded material and the alveolar bone distant from the periapex (HE, 10×)

Studies evaluating the success of endodontically treated teeth showed that overfills delayed the apical repair process^52^ ^,^ ^99^ ^,^ ^100^; the apical limit of obturation can affect the final outcome of endodontic treatment^100^. In addition, the findings observed in histological investigations corroborate these clinical and radiographic studies^52^ (Figure 2 and Figure 3).

Evidence has shown that the type of sealer applied can also affect the repair process. Suzuki, et al.^99^ (2011) investigated the effects of endomethasone as a filling material in canine teeth and found that none of the filling limits (inferior or above the apical foramen) promoted the complete repair of the periapical tissue. Furthermore, the presence of an intense inflammatory infiltrate was also observed in the cases of overfilling. Similar results were also obtained with EndoRez when evaluated as a filling material in canine teeth^100^, therefore corroborating the studies that showed high toxicity^8^ and the presence of moderate to severe inflammatory reactions in intraosseous implants^96^.

However, even the use of biocompatible materials, such as MTA, when used as filling beyond the limit of the apical foramen, showed unsatisfactory results^52^; this suggested that overfilling should be avoided.

### Expansion of the apical foramen

Root canal cleaning with the use of flexible files is recommended for the endodontic treatment of necrotic teeth; additionally, the apical constriction is maintained. This procedure, which is also referred to as apical patency, is common; it prevents the compaction of dentin chips into the foramen and helps the local elimination of microorganisms that inhibit the process of repair following endodontic treatment. However, studies on canine teeth with periapical lesions showed that optimal results were obtained when the apical foramen was expanded to a greater extent than the patency instrument^6^.

The enlargement of the apical foramen affects the healing of chronic periapical lesions^6^. According to some authors, the compaction of debris into the apical third of the root canal should be removed mechanically^106^, while others believe that it can be removed with the use of abundant irrigation^52^, and that the infection could be additionally eliminated with the use of an intracanal dressing^54^. Moreover, the enlargement of the foramen appeared to hinder the apical seal^39^.

Apical patency is an important factor determining the success of endodontic treatment; nonetheless, further research concerning the enlargement of the foramen is required.

### Systemic factors that interfere in the repair process

The repair of apical periodontitis in endodontically treated teeth depends on different therapeutic factors and clinical conditions^19^ ^,^ ^65^ ^,^ ^89^. Seltzer^90^ (1988) correlates the local and systemic factors affecting the endodontic repair process, and suggests that the failure of the endodontic treatment may be beyond the dentist's control. Local factors include: infection; hemorrhage, tissue injury; occlusion of the blood supply; and presence of foreign bodies. Systemic factors include: nutrition; stress; state of chronic debilitation; hormones; vitamin intake; dehydration; and age. Therewith, this review also aims to discuss the association between some systemic conditions, such as diabetes mellitus, hypertension, and osteoporosis, and the periapical healing process of endodontically treated teeth.

### Chronic conditions

#### Diabetes

Diabetes mellitus (DM) is a metabolic disorder characterized by hyperglycemia resulting from a defect in the secretion of insulin and/or the impairment of its action^3^. Studies have been emphasizing the relationship between DM and oral diseases^29^ ^,^ ^48^ ^,^ ^65^ ^,^ ^89^. Fouad^25^ (2003) showed that DM may be a modulating factor of endodontic infections, and that it may compromise the healing process of periapical tissues.

The association between periodontal disease and the incidence of DM has been reported in some studies^48^; however, few studies have suggested that the incidence of DM was comorbid with endodontic disease. Cintra, et al.^12^ (2014) studied the influence of apical periodontitis and periodontal disease by examining the concentration of glycosylated hemoglobin (HbA1c) in normoglycemic and hyperglycemic rats. They observed that the incidence of endodontic infections that were either isolated or associated with periodontal disease had affected the glycemic control, especially in diabetic rats. This resulted in an increase in both blood glucose and HbA1c levels.

In another study^14^, the relationship between the blood profile and the histological findings of the cases involving apical periodontitis and periodontal disease associated with diabetes was examined. In the presence of oral infection, the authors observed an increase in the number of neutrophils, lymphocytes, and leukocytes, as well as significant statistical difference between the diabetics and the diabetics with endodontic and periodontal infection. Histological findings showed an exacerbation of inflammation, with the consequent increase in inflammatory cell infiltration and bone resorption in diabetic rats.

The relationship between endodontic infections and the interaction with systemic diseases is not clear. Hyperglycemia elevates the levels of systemic inflammatory markers^48^ and alters the various functions of the immune system^91^ ^,^ ^102^, including the release of inflammatory mediators. Cintra, et al.^13^ (2014) correlated the serum levels of interleukin-17 (IL-17) and the infiltration of neutrophils in the presence of apical periodontitis and/or periodontal disease in diabetic rats. They found that the comorbidity of both diseases increased the serum levels of IL-17 regardless of the diabetic condition. Furthermore, an increase in the neutrophil population was observed in diabetic rats.

Hyperglycemia resulting from DM promoted molecular and cellular effects that can predispose individuals to systemic complications, such as the dysfunction and failure of various organs^3^. Nephropathy is one of the chronic complications of DM, and a possible relationship to periodontal disease has been reported^49^.

Based on a previous study^15^, a relationship between the periapical lesions and/or the presence of periodontal disease and the serum creatinine levels in diabetes was also suggested. An increase in creatinine levels was observed in diabetic rats; the levels were elevated in the presence of oral infection. This suggested that a high glycemic increase may predispose individuals to renal disorders. An additional investigation was performed to evaluate the triglyceride and cholesterol levels in diabetic rats. The relationship between these factors and the incidence of pulpal and periodontal diseases was then examined; it was observed that the isolated cases of periodontal infection, or its association with endodontic infections, led to an increase in the triglyceride levels of diabetic rats. However, the difference in the cholesterol levels was not significant^16^.

**Figure 4:**
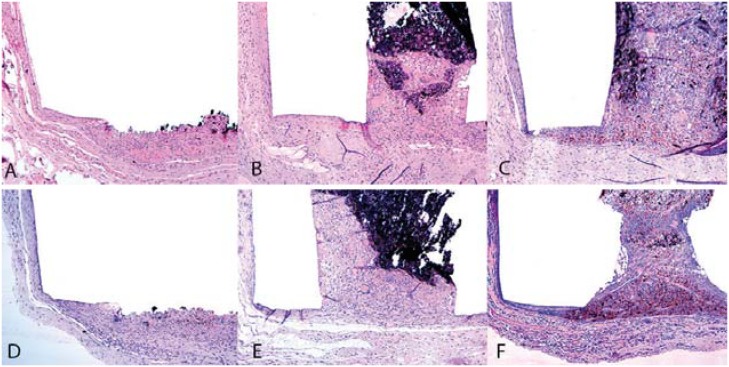
Tissue response of the subcutaneous tissue on the 30^th^ day under normal (A, B, C) and diabetic (D, E, F) conditions. Use of Gray MTA Angelus^®^ (A) and MTA Fillapex^®^ (B): in both materials, a mild inflammatory response with the infiltration of macrophages and lymphocytes was evident (hematoxylin-eosin, 10× magnification); Sealapex^®^ (C): thick, fibrous capsule formation and a moderate inflammatory cell infiltration (hematoxylin-eosin, 10× magnification); Gray MTA Angelus^®^ (D): presence of a moderate inflammatory infiltration of macrophages and lymphocytes (hematoxylin-eosin, 10× magnification); MTA Fillapex^®^ (E): presence of a mild inflammatory infiltration of macrophages and lymphocytes after 30 days (hematoxylin-eosin, 10× magnification); Sealapex^®^ (F): thick, fibrous capsule formation and moderate inflammatory cell infiltration (hematoxylin-eosin, 10× magnification)

Alterations in the health of the skeletal system were also associated with diabetes^113^. The control of inflammation is essential in the bone repair process^4^. Systemic inflammation, which is promoted by DM, can lead to a prolonged healing time in the case of fractures^17^. Previous studies^38^ ^,^ ^41^ evaluated the mineralization capacity and tissue response of MTA cement and two endodontic sealers, MTA Fillapex and Sealapex, using a diabetic model. The tissue response of these sealers was unaffected by the incidence of DM (Figure 4).

#### Hypertension

Hypertension is a chronic disorder that is characterized by an increased peripheral vascular resistance to blood flow. It is attributable to vascular remodeling, and elevates blood pressure in arteries^111^.

The relationship between hypertension and calcium loss in bones has been shown in clinical and experimental studies^32^ ^,^ ^109^. Likewise, the alteration in the activity and differentiation of bone cells observed in patients has been related to the incidence of hypertension^60^. It has been correlated with elevated blood pressure, incidence of dental problems (such as periodontal disease^56^ ^,^ ^62^), high rate of implant loss due to defects in the process of osseointegration^2^, and difficulties in bone healing following tooth extractions^69^.

Periodontal disease and chronic apical periodontitis showed similar inflammatory processes. Furthermore, patients with systemic diseases may have a reduced resistance to bacterial infection and tissue repair^85^. Periapical lesions occur as an inflammatory response to infection and, along with hypertension, can lead to vascular injury and inflammation^70^.

Martins, et al.^70^ (2016) evaluated the tissue response and mineralization capacity of MTA cement in hypertensive rats. The results showed that hypertensive rats had an intense inflammatory reaction and were characterized by a decreased mineralization rate compared to normotensive rats; this suggested that the comorbidity of hypertension was able to impair the tissue response and the mineralization ability of MTA.

The hypertensive state can be a risk factor for periodontitis and periapical lesions. However, further research linking hypertension and periapical lesion healing is required.

#### Menopause/Osteoporosis

The longer life expectancy of elderly individuals raises a concern for both the quality of life and the prevention of age-related diseases. In this context, an increase in the prevalence of bone fracture because of advanced age has been observed^11^, particularly in post-menopausal women; this is a result of the decreased concentration of estrogen.

Osteoporosis and apical periodontitis are two diseases that involve bone resorption. Studies have shown a significant correlation between these two diseases^98^ ^,^ ^112^. Xiong, et al.^112^ (2007) showed that the condition of apical periodontitis is aggravated by an estrogen deficiency. Low estrogen concentration also promoted an increase in the resorptive activity of the alveolar process in rats^46^ ^,^ ^112^.

Hormone replacement therapies (or the use of stabilizing substances for the process of resorption) aim to reduce the frequency of fractures and to prevent further loss of mature bone^9^. This includes the use of bisphosphonate^9^ ^,^ ^28^. Raloxifene, which is an example of bisphosphonate, is used in the treatment and prevention of osteoporosis in postmenopausal women^27^. Investigations using ovariectomized rats showed that this treatment also prevented bone loss and suppressed its formation. This resulted in reduced bone remodeling^45^ ^,^ ^46^.

In addition, the relationship between the incidence of apical periodontitis in ovariectomized rats, with or without the treatment of Raloxifene, suggested that a deficiency in estrogen concentrations enhanced the progression of periapical lesions^45^ ^,^ ^46^.

## Conclusion

In summary, the repair process of endodontically treated teeth depends not only on the adoption of the correct clinical approaches to promote a better RCT (such as use of good irrigant solution, intracanal dressing, and root canal filling), but also on systemic factors (such as chronic diseases, hormones, and age) that can change the host's immune defenses and interfere in the outcome of root canal treatment and in the healing process.
